# Supplementary material for: Multi‐dataset identification of innovative feature genes and molecular mechanisms in keratoconus
Source: J Cell Mol Med. 2024 Sep 19;28(18):e70079. doi: 10.1111/jcmm.70079 (PMC11412914; doi:10.1111/jcmm.70079)
Supplement: Supplementary file 2 — Material S2. [file JCMM-28-e70079-s002.zip › 7.GSEA analysis/5.RNF217-AS1/KEGG_GLYCOSYLPHOSPHATIDYLINOSITOL_GPI_ANCHOR_BIOSYNTHESIS.html]

Details for gene set KEGG\_GLYCOSYLPHOSPHATIDYLINOSITOL\_GPI\_ANCHOR\_BIOSYNTHESIS[GSEA]

|  || Dataset | RNF217-AS1.group.cls#h\_versus\_l |
| Phenotype | group.cls#h\_versus\_l |
| Upregulated in class | l |
| GeneSet | KEGG\_GLYCOSYLPHOSPHATIDYLINOSITOL\_GPI\_ANCHOR\_BIOSYNTHESIS |
| Enrichment Score (ES) | -0.458807 |
| Normalized Enrichment Score (NES) | -1.4284179 |
| Nominal p-value | 0.1434263 |
| FDR q-value | 0.33396304 |
| FWER p-Value | 0.845 |
Table: GSEA Results Summary

  

Fig 1: Enrichment plot: KEGG\_GLYCOSYLPHOSPHATIDYLINOSITOL\_GPI\_ANCHOR\_BIOSYNTHESIS      
 Profile of the Running ES Score & Positions of GeneSet Members on the Rank Ordered List

  

| SYMBOL | TITLE | RANK IN GENE LIST | RANK METRIC SCORE | RUNNING ES | CORE ENRICHMENT || 1 | PIGL | na | 4668 | 0.228 | -0.0470 | No |
| 2 | PGAP1 | na | 8922 | 0.107 | -0.1287 | No |
| 3 | PIGA | na | 13844 | 0.032 | -0.2581 | No |
| 4 | PIGY | na | 15903 | 0.010 | -0.3137 | No |
| 5 | DPM2 | na | 19802 | -0.028 | -0.4153 | No |
| 6 | PIGG | na | 20465 | -0.035 | -0.4211 | No |
| 7 | PIGK | na | 20571 | -0.036 | -0.4105 | No |
| 8 | PIGC | na | 21463 | -0.045 | -0.4190 | No |
| 9 | PIGH | na | 21571 | -0.046 | -0.4044 | No |
| 10 | PIGF | na | 23459 | -0.068 | -0.4329 | Yes |
| 11 | PIGU | na | 23538 | -0.069 | -0.4088 | Yes |
| 12 | PIGO | na | 23838 | -0.073 | -0.3895 | Yes |
| 13 | PIGN | na | 23889 | -0.074 | -0.3628 | Yes |
| 14 | PIGP | na | 24013 | -0.075 | -0.3374 | Yes |
| 15 | PIGB | na | 25262 | -0.095 | -0.3371 | Yes |
| 16 | PIGS | na | 25382 | -0.097 | -0.3035 | Yes |
| 17 | PIGW | na | 25447 | -0.098 | -0.2680 | Yes |
| 18 | PIGT | na | 25752 | -0.103 | -0.2374 | Yes |
| 19 | PIGX | na | 26179 | -0.110 | -0.2075 | Yes |
| 20 | GPAA1 | na | 26292 | -0.112 | -0.1677 | Yes |
| 21 | PIGV | na | 27159 | -0.129 | -0.1432 | Yes |
| 22 | PIGM | na | 28712 | -0.168 | -0.1234 | Yes |
| 23 | PIGZ | na | 29952 | -0.205 | -0.0808 | Yes |
| 24 | PIGQ | na | 30716 | -0.231 | -0.0144 | Yes |
| 25 | GPLD1 | na | 32727 | -0.339 | 0.0577 | Yes |
Table: GSEA details [plain text format]

  

Fig 2: KEGG\_GLYCOSYLPHOSPHATIDYLINOSITOL\_GPI\_ANCHOR\_BIOSYNTHESIS      
 Blue-Pink O' Gram in the Space of the Analyzed GeneSet

  

Fig 3: KEGG\_GLYCOSYLPHOSPHATIDYLINOSITOL\_GPI\_ANCHOR\_BIOSYNTHESIS: Random ES distribution      
 Gene set null distribution of ES for **KEGG\_GLYCOSYLPHOSPHATIDYLINOSITOL\_GPI\_ANCHOR\_BIOSYNTHESIS**
